# Towards reducing the immunogenic potential of wheat flour: omega gliadins encoded by the D genome of hexaploid wheat may also harbor epitopes for the serious food allergy WDEIA

**DOI:** 10.1186/s12870-018-1506-z

**Published:** 2018-11-21

**Authors:** Susan B. Altenbach, Han-Chang Chang, Annamaria Simon-Buss, You-Ran Jang, Sandra Denery-Papini, Florence Pineau, Yong Q. Gu, Naxin Huo, Sun-Hyung Lim, Chon-Sik Kang, Jong-Yeol Lee

**Affiliations:** 1 USDA-ARS Western Regional Research Center, 800 Buchanan Street, Albany, CA 94710 USA; 2 National Institute of Agricultural Sciences, RDA, Jeonju, 54874 South Korea; 3 UR1268 Biopolymers, Interactions, Assemblies, Institut National de la Recherche Agronomique, Rue de la Géraudière, F-44316 Nantes, France; 4 National Institute of Crop Science, RDA, Jeonju, 55365 South Korea

**Keywords:** Food allergy, Omega-5 gliadins, Proteomics, Reduced immunogenic potential, Wheat flour

## Abstract

**Background:**

Omega-5 gliadins are a group of highly repetitive gluten proteins in wheat flour encoded on the 1B chromosome of hexaploid wheat. These proteins are the major sensitizing allergens in a severe form of food allergy called wheat-dependent exercise-induced anaphylaxis (WDEIA). The elimination of omega-5 gliadins from wheat flour through biotechnology or breeding approaches could reduce the immunogenic potential and adverse health effects of the flour.

**Results:**

A mutant line missing low-molecular weight glutenin subunits encoded at the *Glu-B3* locus was selected previously from a doubled haploid population generated from two Korean wheat cultivars. Analysis of flour from the mutant line by 2-dimensional gel electrophoresis coupled with tandem mass spectrometry revealed that the omega-5 gliadins and several gamma gliadins encoded by the closely linked *Gli-B1* locus were also missing as a result of a deletion of at least 5.8 Mb of chromosome 1B. Two-dimensional immunoblot analysis of flour proteins using sera from WDEIA patients showed reduced IgE reactivity in the mutant relative to the parental lines due to the absence of the major omega-5 gliadins. However, two minor proteins showed strong reactivity to patient sera in both the parental and the mutant lines and also reacted with a monoclonal antibody against omega-5 gliadin. Analysis of the two minor reactive proteins by mass spectrometry revealed that both proteins correspond to omega-5 gliadin genes encoded on chromosome 1D that were thought previously to be pseudogenes.

**Conclusions:**

While breeding approaches can be used to reduce the levels of the highly immunogenic omega-5 gliadins in wheat flour, these approaches are complicated by the genetic linkage of different classes of gluten protein genes and the finding that omega-5 gliadins may be encoded on more than one chromosome. The work illustrates the importance of detailed knowledge about the genomic regions harboring the major gluten protein genes in individual wheat cultivars for future efforts aimed at reducing the immunogenic potential of wheat flour.

**Electronic supplementary material:**

The online version of this article (10.1186/s12870-018-1506-z) contains supplementary material, which is available to authorized users.

## Background

The gluten proteins are a complex group of 70–100 abundant proteins that confer the unique viscoelastic properties to wheat flour. At the same time, some of these proteins are responsible for human health problems, including food allergies and celiac disease. Among the gluten proteins, the omega gliadins are particularly immunogenic [1]. These proteins comprise 5–10% of total flour protein, depending on the cultivar and the growth conditions of the plant. Omega gliadins consist almost entirely of repetitive sequences with unusually high proportions of glutamine and proline (~ 68–73%) and generally lack cysteine. The proteins are divided into two groups, referred to as omega-5 and omega-1,2 gliadins, that differ in N-terminal sequences and repetitive motifs. Omega-5 gliadins usually begin with SRL and contain multiple copies of FPQQQ and QQIPQQ while omega-1,2 gliadins begin with ARE, ARQ or KEL and contain the repetitive motif PQQPFP. Omega-5 gliadins are encoded at the *Gli-1* locus on the short arm of chromosome 1B in hexaploid wheat and are the major sensitizing allergens in the serious food allergy wheat-dependent exercise-induced anaphylaxis (WDEIA) that occurs in sensitized individuals when the ingestion of wheat is followed by physical exercise [2]. Omega-1,2 gliadins are encoded on chromosomes 1A and 1D and contain immunodominant T-cell stimulatory epitopes involved in celiac disease [3]. Omega gliadins show some of the largest changes among gluten proteins in response to the application of fertilizer or high temperatures during grain development [4–7], likely influencing the immunogenic potential of the flour.

Despite their importance, detailed studies of the omega gliadins have been challenging. Omega-5 gliadins, in particular, are difficult to identify by tandem mass spectrometry (MS/MS) because they contain a limited number of proteolytic cleavage sites. More importantly, there is a lack of complete protein sequences for omega-5 gliadins in databases despite the considerable allelic diversity in these proteins among cultivars. In fact, until recently, only one of more than 100 omega gliadin protein sequences in NCBI, BAE20328, had a predicted molecular weight within the 48.9–51.5 kDa range determined for omega-5 gliadins by mass spectrometry [8]. Four proteins, AJG03093, AJG03080, AJG03079 and CAR82267, are likely missing portions of their repetitive regions and have predicted molecular weights ranging from 36 to 42 kDa, while many other omega-5 gliadin proteins in NCBI are missing sizeable portions of their central repetitive regions [9]. The lack of full-length protein sequences is most likely due to difficulties in cloning their highly repetitive genes [10]. Recently, genomic sequences of regions that harbor prolamin genes from the reference wheat Chinese Spring were assembled and annotated [11] and two additional full-length omega-5 gliadin protein sequences were added to NCBI, omega-B3 (AWK59777) and omega-B6 (AWK59773), corresponding to proteins of 47.7 and 51.5 kDa, respectively.

Arrays of overlapping peptides based on the full-length sequence of BAE20328 were used to identify IgE binding epitopes for WDEIA [12]. The peptides QQFPQQQ, QQIPQQQ, QQSPQQQ and QQSPEQQ were found to be dominant epitopes with amino acids in positions one, four, five, six and seven critical for IgE binding. Interestingly, there are 23 copies of QQFPQQQ and 4 copies of QQIPQQQ in BAE20328, some of which are overlapping, that contribute to the immunogenicity of the protein.

In recent years, a number of strategies have been explored to reduce the levels of the omega-5 gliadins in wheat flour with the goal of reducing the immunogenic potential of the flour. One approach involves selecting genotypes with reduced levels of these proteins. Towards this end, Denery-Papini et al. [13] surveyed flour proteins from a collection of cultivars expressing 13 different omega-5 gliadin alleles for reactivity with IgE from WDEIA patients. Only one cultivar containing a wheat/rye translocation showed low levels of reactivity. However, these translocation lines had poor breadmaking properties due to the replacement of the omega-5 gliadins with the secalins from rye. Another group [14] identified a spontaneous mutation in a line from a cross between a spelt cultivar and a Polish breeding line that resulted in an inactive omega-5 gliadin gene at the *Gli-B1* locus. After crossing this line to another line containing inactive genes at the *Gli-A1* and *Gli-D1* loci, they reported a ~ 30% reduction in immunoreactivity using ELISA. Biotechnology approaches using RNA interference have also been used to reduce the levels of omega-5 gliadins, resulting in transgenic wheat plants with reduced IgE reactivity to sera from WDEIA patients without adverse effects on flour end-use functional properties [15–17].

Recently, the wheat mutant DH20, missing LMW-GS encoded by the B genome, was identified by screening glutenins from a double haploid population derived from a cross between two Korean wheat cultivars, the hard white wheat Keumkang and the soft red wheat Olgeuru [18]. When evaluated in the field over 2 years, DH20 demonstrated good agronomic properties as well as a slightly higher yield than the parental cultivars. In addition, the protein content, SDS sedimentation volume and mixing tolerance of DH20 flour were similar to Keumkang, a leading Korean bread wheat cultivar with good milling quality and medium dough strength. In this paper, we compare the total protein composition of DH20 flour to that of the parental lines and report that DH20 is also missing the major omega-5 gliadins, resulting in a wheat with reduced immunogenic potential. However, the analysis revealed that several minor but highly immunogenic omega-5 gliadins remained in this line. The work highlights some of the challenges faced in using breeding approaches to reduce the immunogenic potential of wheat flour.

## Results

### 2-DE analysis of total flour proteins

**Fig. 1:**
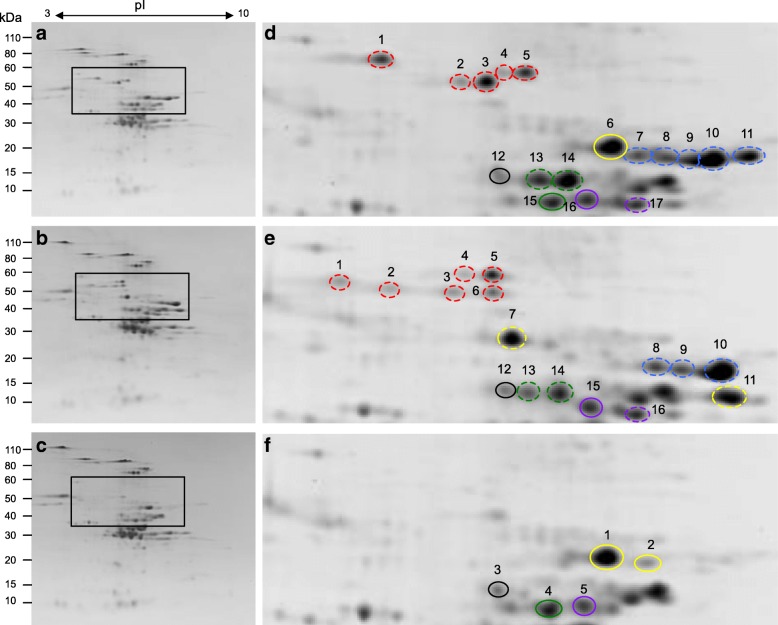
Total flour proteins from parental lines Keumkang (**a**, **d**) and Olgeuru (**b**, **e**) and mutant line DH20 (**c**, **f**). Protein spots within the black boxes in panels (a, b and c) were identified by MS/MS. Proteins circled in red, blue, yellow, purple, green and black in panels (d, e and f) were identified as omega-5 gliadins, s-type LMW-GS, i-type LMW-GS, m-type LMW-GS, gamma gliadins and triticins, respectively. Dashed circles denote proteins identified in at least one of the parental lines but absent in the mutant line. MS/MS data are summarized in Table 1 and Additional file 1

Analyses of total flour proteins from the parental lines Keumkang and Olgeuru and the mutant line DH20 by two-dimensional gel electrophoresis (2-DE) revealed that a number of gluten proteins between ~ 34 and 55 kDa are missing in the mutant (Fig. 1). As documented previously, these include a number of LMW-GS encoded by the *Glu-B3* locus [18]. Spots 7–11 in Keumkang (Fig. 1d), identified by MS/MS as s-type LMW-GS ACA63869 and ACA63865 (Table 1, Additional file 1), and spots 8–10 in Olgeuru (Fig. 1e), identified as s-type LMW-GS ACA63864 and ACA63868 (Table 1, Additional file 1), are missing in the mutant line DH20 (Fig. 1f). These proteins have the N-terminal sequences SHIPGLERP and differ by only a few amino acids. In addition, DH20 is missing an m-type LMW-GS also encoded by the *Glu-B3* locus. The LMW-GS ACA63871 was identified in spot 17 in Keumkang (Fig. 1d) and the LMW-GS ACP27643 was identified in spot 16 in Olgeuru (Fig. 1e). These two proteins begin with the N-terminal sequences METSHIP and differ by only 5 amino acids. The DH20 mutant contained the i-type LMW-GS AAS10189 (spot 1 in Fig. 1f) contributed by the Keumkang parent (spot 6 in Fig. 1d), confirming the presence of the *Glu-A3c* allele in the mutant line. AAS10189 was also identified in spot 2 in DH20 and was a minor component of Spot 7 in Keumkang (Fig. 1d, Additional file 1). All three lines contained the same m-type LMW-GS ABC84361 beginning with the N-terminal sequence METSRV that is contributed by the *Glu-D3a* allele (spot 16 in Keumkang (Fig. 1d), spot 15 in Olgeuru (Fig. 1e) and spot 5 in DH20 (Fig. 1f)). These spots serve as references among the gels.

**Table 1:** Identification of 2-DE spots from parental and mutant lines by MS/MS

Cultivar	Spot #	Predominant Protein	Accession Number	# Exclusive Unique Peptides	# Spectra	% Coverage
Keumkang	1	omega-5 gliadin	CAR82267	16	47	29
Keumkang	2	omega-5 gliadin	BAE20328	16	48	25
Keumkang	3	omega-5 gliadin	BAE20328	28	59	38
Keumkang	4	omega-5 gliadin	BAE20328	6	12	19
Keumkang	5	omega-5 gliadin	BAE20328	15	41	40
Keumkang	6	LMW-GS (i-type)^a^	AAS10189	59	180	81
Keumkang	7	LMW-GS (s-type)^b^	ACA63869	67	191	73
Keumkang	8	LMW-GS (s-type)^b^	ACA63869	64	211	72
Keumkang	9	LMW-GS (s-type)^b^	ACA63869	90	305	76
Keumkang	10	LMW-GS (s-type)^b^	ACA63869	130	443	88
Keumkang	11	LMW-GS (s-type)^b^	ACA63865	90	313	78
Keumkang	12	triticin	ACB41345	30	74	29
Keumkang	13	gamma gliadin	Bu-5^d^	53	177	60
Keumkang	14	gamma gliadin	Bu-5^d^	47	164	61
Keumkang	15	gamma gliadin	AGJ50340	46	164	62
Keumkang	16	LMW-GS (m-type)^c^	ABC84361	66	179	74
Keumkang	17	LMW-GS (m-type)^c^	ACA63871	70	357	84
Olgeuru	1	omega-5	AIU64835	7	26	48
Olgeuru	2	omega-5	AII26682	6	15	17
Olgeuru	3	omega-5	AII26682	4	15	16
Olgeuru	4	omega-5	CAR82266	6	13	25
Olgeuru	5	omega-5	BAE20328	36	75	45
Olgeuru	6	omega-5	AII26682	9	25	34
Olgeuru	7	LMW-GS (i-type)^a^	BAB78764	60	175	77
Olgeuru	8	LMW-GS (s-type)^b^	ACA63864	40	110	54
Olgeuru	9	LMW-GS (s-type)^b^	ACA63868	44	124	62
Olgeuru	10	LMW-GS (s-type)^b^	ACA63864	92	310	73
Olgeuru	11	LMW-GS (i-type)^c^	ACY08811	47	111	69
Olgeuru	12	triticin	ACB41345	24	60	27
Olgeuru	13	gamma gliadin	AGO17716	23	51	45
Olgeuru	14	gamma gliadin	AGO17716	31	74	57
Olgeuru	15	LMW-GS (m-type)^c^	ABC84361	60	164	74
Olgeuru	16	LMW-GS (m-type)^c^	ACP27643	23	118	51
DH20	1	LMW-GS (i-type)^a^	AAS10189	104	310	83
DH20	2	LMW-GS (i-type)^a^	AAS10189	30	98	70
DH20	3	triticin	ACB41345	39	129	37
DH20	4	gamma gliadin	AGJ50340	49	161	58
DH20	5	LMW-GS (m-type)^c^	ABC84361	60	164	74

^a^refers to LMW-GS that begin with the N-terminal sequence ISQQQ

^b^refers to LMW-GS that begin with the N-terminal sequence SHIP

^c^refers to LMW-GS that begin with M

^d^best match is to a sequence from cv. Butte 86 reported in [19]

Most notably, the omega-5 gliadins were absent from the mutant line. In Keumkang, spots 1–5 were identified as omega-5 gliadins CAR82267 or BAE20328 (Fig. 1d, Table 1, Additional file 1) while spots 1–6 in Olgeuru were identified as CAR82267, BAE20328 or the partial sequences AIU64835 or AII26682 (Fig. 1e, Table 1, Additional file 1). All proteins in these regions of the 2-D gels were missing in DH20 (Fig. 1f).

In addition, several gamma gliadins were missing from DH20, including spots 13 and 14 from Keumkang (Fig. 1d) and 13 and 14 from Olgeuru (Fig. 1e). Both spots in Keumkang were identified as Bu-5, a gamma gliadin from the U.S. cultivar Butte 86 [19], and both spots in Olgeuru were identified as AGO17716. The two proteins differ by only 8 amino acids. Spots 15 in Keumkang and 4 in DH20, identified as gamma gliadin AGJ50340 along with spots 12 in both Keumkang and Olgeuru and spot 3 in DH20, identified as triticin, serve as reference spots among the gels.

### PCR analysis of parental and mutant lines

**Fig. 2:**
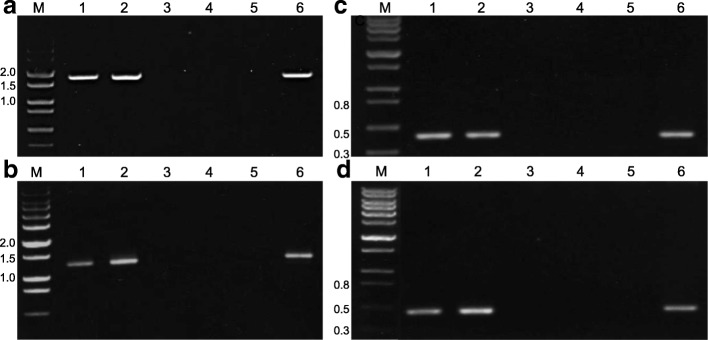
PCR analysis with primers specific for LMW-GS encoded at the *Glu-B3* locus (**a**), omega-5 gliadins (**b**), and repeat junction primers 19S-1.3-2 (**c**) and 143E-1-600 (**d**) located at the ends of a 5.8 Mb region of chromosome 1B in Chinese Spring. In each panel, genomic DNA from Keumkang (1), Olgeuru (2), DH20 (3), N1BT1A (4) and N1BT1D (5) nullisomic tetrasomic lines of Chinese Spring or Chinese Spring (6) was amplified. The sizes of molecular weight markers in kb are shown in lane M in each panel. Primer sequences are shown in Table 2

PCR analysis confirmed that a number of genes were deleted in DH20 (Fig. 2). PCR using primers specific for the *Glu-B3–4* LMW-GS gene (Table 2) yielded a fragment of ~ 1600 bp from genomic DNA of Keumkang, Olgeuru, and the reference wheat Chinese Spring, but not from the DH20 mutant or the N1BT1A and N1BT1D nullisomic tetrasomic lines of Chinese Spring (Fig. 2a). Likewise, the expected 1200 bp fragment was obtained from Keumkang, Olgeuru and Chinese Spring using primers specific for the omega-5 gliadin gene (Table 2), but not from DH20 or the N1BT1A and N1BT1D nullisomic tetrasomic lines of Chinese Spring (Fig. 2b), confirming the location of the omega-5 gliadin genes on chromosome 1B and the deletion of these genes in DH20.

**Table 2:** PCR primers and amplification conditions used in this study

Target	Primer set	Sequence (5′ - 3′)	Amplicon size	PCR conditions	Reference
*GluB3–4* LMW-GS	LB4F	CACCCTATACAAGGTTCCAAAAT	1636	94 °C 60s, 60 °C 60s, 72 °C 60s	[27]
	LB4R	TATTTCCATAATTTAAACTAGTTTGT			
Omega-5 gliadin	O5GF	AGTAGGCTGCTAAGCCCTAGA	1212	94 °C 30s, 60 °C 45s, 72 °C 60s	This study
	O5GR	ATATTGTTGGTATGGGGAAGG			
Chromosome 1B Repeat Junction Primers	19S-1.3-2F	CGGGACTCATTGAAGAATCC	418	94 °C 30s, 55 °C 45s, 72 °C 60s	This study
	19S-1.3-2R	ACTAAAGTAGCGATTCAAATCCTC			
Chromosome 1B Repeat Junction Primers	143E-1-600F	CAACGACATATTCTAACCTCCACA	482	94 °C 30s, 60 °C 45s, 72 °C 60s	This study
	143E-1-600R	ACATACATACATCACTGACCGACTG			
Omega-D4	OD4-F1	ACTAGGCAACTAAGCCCTAGA	1037	95 °C 30s, 60 °C 30s, 72 °C 45s	This study
	OD4-R1	GCTTCTTGCGATTGTTGTTGG			

To determine the extent of the deletion in DH20, repeat junction primers were selected based on the recently published genomic sequence of a 6.5 Mb region of chromosome 1B from Chinese Spring that contains LMW-GS, omega-5 gliadin and gamma gliadin genes [11] (Table 2). These primers amplified the expected size fragments from the parental lines Keumkang and Olgeuru as well as Chinese Spring, but did not amplify DH20 or the N1BT1A and N1BT1D nullisomic tetrasomic Chinese Spring lines, suggesting that, at a minimum, a 5.8 Mb region of the 1B chromosome had been deleted in DH20.

### Immunogenic potential of parental and mutant lines

**Fig. 3:**
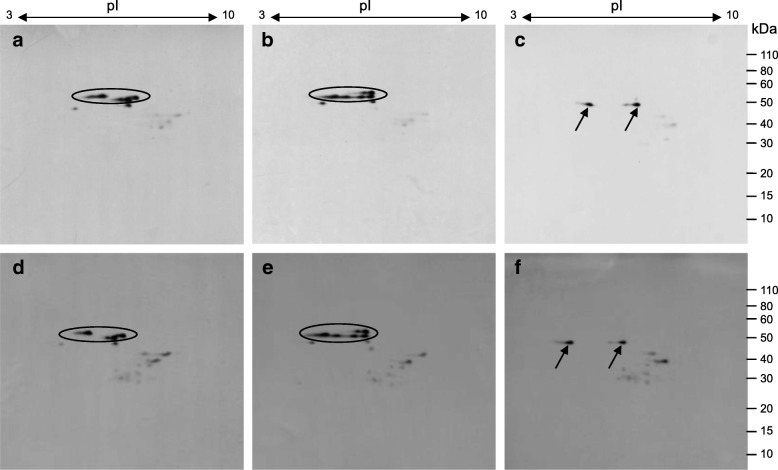
Reactivity of serum from WDEIA patients with total flour proteins from the parental cultivars Keumkang (**a**, **d**), Olgeuru (**b**, **e**) or the mutant DH20 (**c**, **f**). Serum was from patient #4 (**a**-**c**) and patient #5 (**d**-**f**) described in [16]. Positions of protein spots identified as omega-5 gliadins in the parental lines that are absent in the mutant line are shown with an oval. Arrows in panels (**c** and **f**) point to unidentified protein spots that are reactive in both the mutant and the parental lines

Since the omega-5 gliadins are the major sensitizing proteins in the serious food allergy WDEIA, the immunogenic potential of DH20 was evaluated by 2-D immunoblot analysis using sera from patients with verified cases of WDEIA. Patient sera were characterized previously by ELISA using individual gluten protein fractions and by 2D immunoblot analysis [16]. In the two parental lines, proteins identified as omega-5 gliadins showed the greatest amounts of IgE reactivity (Fig. 3a, b, d, e). In addition, there was some reactivity with two protein spots with molecular weights slightly less than the omega-5 gliadins as well as with some proteins in the LMW-GS and alpha gliadin regions of the 2D gels. IgE reactivity was reduced markedly in DH20 (Fig. 3c, f). There was no reactivity in the region of the omega-5 gliadins. However, the two protein spots just below the omega-5 gliadin region, indicated with arrows in Fig. 3, showed reactivity with the sera from both WDEIA patients. These spots correspond to very minor proteins on the Coomassie-stained gels (Fig. 1).

**Fig. 4:**
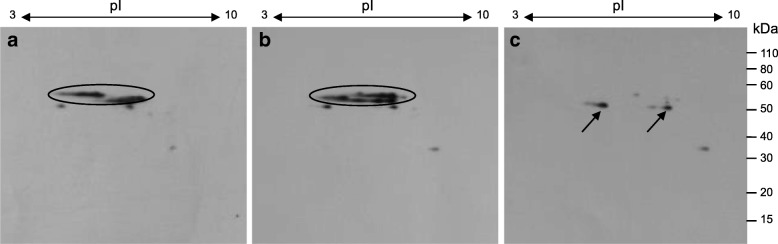
Reactivity of a monoclonal antibody made to the N-terminal sequence of omega-5 gliadin with total flour proteins from (**a**) Keumkang (**b**) Olgeuru or (**c**) DH20. Positions of protein spots identified as omega-5 gliadins in the parental lines that are absent in the mutant line are shown with an oval. Arrows in the mutant line in panel (**c**) point to unidentified protein spots that are reactive in both the mutant and the parental lines

Immunoblots were also reacted with a monoclonal antibody made against the N-terminal sequence of omega-5 gliadin (Fig. 4). As expected, the antibody reacted strongly with the proteins identified as omega-5 gliadins in Keumkang (spots 1–5 in Fig. 1d) and Olgeuru (spots 1–6 in Fig. 1e). However, the antibody also reacted with the two minor protein spots just below the identified omega-5 gliadins in the parental lines as well as in the mutant line (Fig. 4), suggesting that these spots also correspond to omega-5 gliadins. Minor reactivity with a more basic protein of about 35 kDa was also observed in all of the lines, but was difficult to align with a specific spot on the Coomassie stained gels.

### Arrangement of omega gliadin genes in cv. Chinese spring

**Fig. 5:**
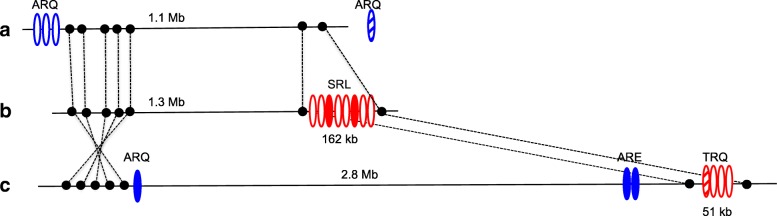
Genomic arrangement of omega gliadin genes on chromosomes 1A (**a**), 1B (**b**) and 1D (**c**) of Chinese Spring summarized from [11]. The positions of ancestral genes found on all three chromosomes are indicated with black dots. Genes with repetitive motifs characteristic of omega-5 and omega-1,2 gliadins are shown in red and blue ovals, respectively. Predicted N-terminal sequences of the encoded proteins are shown above each group of genes. Genes that encode full length omega gliadins are denoted by solid ovals, pseudogenes by open ovals and truncated genes by hatched ovals. If expressed, truncated genes are predicted to encode proteins missing a portion of the COOH terminus. The truncated gene on chromosome 1A is located outside the 5.3 Mb region that was annotated

To investigate the origin of the minor reactive proteins in DH20, the arrangement of omega gliadin genes in the reference wheat Chinese Spring was examined in detail. Of the nineteen omega gliadin genes annotated in Chinese Spring [11], four are located on chromosome 1A, eight on 1B and seven on 1D (Fig. 5). Repetitive motifs of seven genes are characteristic of omega-1,2 gliadins while those of 12 genes are characteristic of omega-5 gliadins. A cluster of eight omega-5 gliadin genes are located within a 162 kb region of chromosome 1B that lies between two ancestor genes common to all three genomes as well as *Brachypodium*, rice and sorghum [20]. Six of these are pseudogenes with various mutations that introduce frameshifts and/or premature stop codons into the coding regions. Interspersed among the pseudogenes are omega-B3 and omega-B6 encoding complete omega-5 gliadins. Interestingly, four other omega-5 gliadin genes are located on a 51 kb region between the orthologous ancestor genes on chromosome 1D (Fig. 5). Three of these are obvious pseudogenes while one, omega-D4, contains a single base deletion 50 bp upstream from the stop codon. If expressed, omega-D4 would encode a protein of 44.4 kDa with an altered C-terminus.

**Fig. 6:**
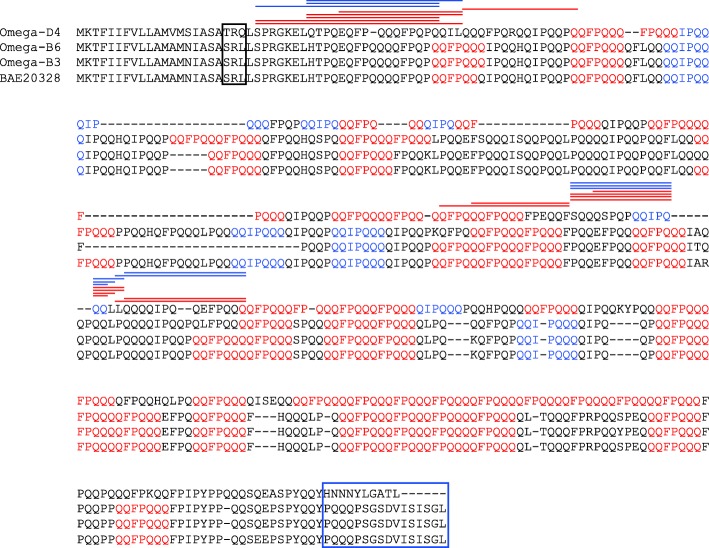
Sequence comparison of omega-5 gliadins encoded by the B and D genomes in Chinese Spring with the full-length omega-5 gliadin BAE20328. N- and C-terminal sequences are shown in black and blue boxes, respectively. Within each protein sequence, the dominant WDEIA epitopes QQFPQQQ and QQIPQQQ are shown in red and blue, respectively. Lines above the protein sequence highlight peptides unique to omega-D4 that were identified by MS/MS in protein spots from DH20 reacting with sera from WDEIA patients. Red lines highlight peptides identified in the more basic protein while blue lines highlight peptides identified in the more acidic protein. MS/MS data are shown in Additional file 1

The amino acid sequences of the omega-5 gliadin proteins encoded by the B genome in Chinese Spring (omega-B3 and omega-B6) were compared to that of omega-D4 (Fig. 6). The only other full-length omega-5 gliadin sequence in NCBI, BAE20328, was also included in the analysis. Omega-B3 differs from BAE20328 by two amino acid substitutions and a 28 amino acid deletion while omega-B6 has six amino acid substitutions and a five amino acid insertion. In comparison, the sequence of omega-D4 is quite different from BAE20328. The N-terminal sequences of the B-encoded proteins and BAE20328 are SRL while that of omega-D4 is TRQ. Additionally, the last 16 amino acids at the C-termini of omega-B3, omega-B6 and BAE20328 are identical while nine of the last 16 amino acids are altered in omega-D4 and the last six amino acids are missing. Despite the smaller size of omega-D4, the protein contains 6 to 8 more copies of the dominant WDEIA epitopes than BAE20328 or the Chinese Spring B-encoded omega-5 gliadins (Table 3). Omega-D4 contains 27 copies of QQFPQQQ and 6 copies of QQIPQQQ. Notably, more than 90% of these epitopes overlap with other epitopes. In fact, there are two regions of the protein where two epitopes overlap, three regions where four epitopes overlap, one region where five epitopes overlap, and one region where nine epitopes overlap. In comparison, only 52–56% of epitopes in B-encoded omega-5 gliadins overlap and the regions of overlap are shorter. BAE20328 and omega-B3 each have only two regions where two epitopes overlap, two regions where 3 epitopes overlap and one region where four epitopes overlap while omega-B6 has four regions where two epitopes overlap, one region where three epitopes overlap and one region where four epitopes overlap (Fig. 6, Table 3).

**Table 3:** Characteristics of omega-5 gliadins

Omega-5 Gliadin	Predicted MW (kDa)	Predicted pI	# WDEIA epitopes		# Overlapping epitopes
			QQFPQQQ	QQIPQQQ	
BAE20328	51.0	6.26	23	4	14
Omega-B3 (AWK59777)	47.7	6.16	22	3	14
Omega-B6 (AWK59773)	51.5	6.01	23	4	15
Omega-D4	44.4	6.34	27	6	30

### Identification of minor immunogenic proteins in DH20

The two minor unidentified proteins that reacted with WDEIA sera in DH20 were excised from 2-D gels, digested with chymotrypsin, thermolysin or trypsin and analyzed by MS/MS. For analysis of spectral data, the sequences of gluten protein genes from Chinese Spring were added to the database used for interrogation. Peptides unique to omega-D4 were identified in spots corresponding to both of the reactive proteins (Fig. 6, Additional file 1), suggesting that these proteins are encoded by the D genome.

**Fig. 7:**
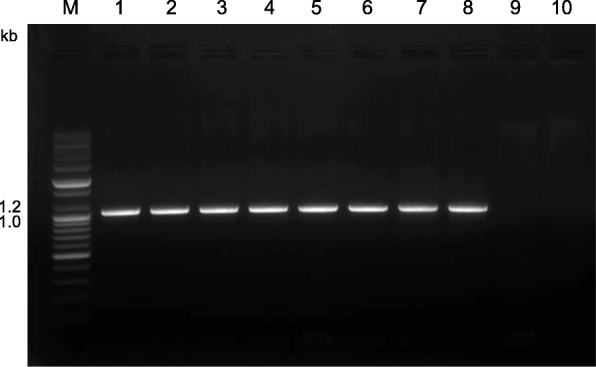
PCR analysis with primers specific for the omega-D4 gene from Chinese Spring. Genomic DNA from Keumkang (1), Olgeuru (2), DH20 (3), Chinese Spring (4) and nullisomic tetrasomic lines of Chinese Spring N1AT1B (5), N1AT1D (6), N1BT1A (7), N1BT1D (8), N1DT1A (9) and N1DT1B (10) was amplified. The sizes of molecular weight markers in kb are shown in lane M

PCR analysis of genomic DNA with primers specific for the Chinese Spring omega-D4 gene (Table 2) demonstrated the presence of omega-D4 in both of the parental lines as well as in the mutant DH20 (Fig. 7). Amplification of aneuploid lines of Chinese Spring further confirmed the location of omega-D4 on the 1D chromosome. Direct sequencing of the PCR product from DH20 using OD4-F1, OD4-R1 and three internal primers (OD4-F3, CAATTCCCCGAACAACAATTC; OD4-F4, AACAGCAATTCCCTCAACAGC; OD4-F5, CAACAACACCAGTTACCG) yielded a single sequence that differed from omega-D4 by an A to C transition 968 bp from the initiation codon. Since this sequence encodes a protein with a pI of 6.84, it is likely that it corresponds to the more basic reactive protein in the immunoblots. It is not known whether DH20 expresses another omega-5 gliadin gene corresponding to the more acid protein.

## Discussion

Analysis of flour proteins by 2-DE combined with MS/MS revealed that s-type and m-type LMW-GS encoded by the B genome as well as omega-5 gliadins and several gamma gliadins are absent in the mutant DH20. It is well known that the LMW-GS genes are tightly linked to gamma and omega gliadin genes on the short arms of the group 1 homoeologous chromosomes in hexaploid wheat. A relatively new group of gluten proteins, the delta gliadins, are also encoded in this region [21]. Recent efforts in genome sequencing revealed the structural organization of these groups of genes in the reference wheat Chinese Spring and identified the sequences of 4 LMW-GS, 4 gamma gliadins, 2 delta gliadins and 3 omega gliadins located on a 5.3 Mb region of the 1A chromosome, 4 LMW-GS, 6 gamma gliadins, 1 delta gliadin and 8 omega gliadins located on a 6.5 Mb region of the 1B chromosome, and 7 LMW-GS, 4 gamma gliadins, 2 delta gliadins and 7 omega gliadins located on a 5.6 Mb region of the 1D chromosome [11]. Amplification of genomic DNA from the parental lines and the mutant line DH20 with primers for omega-5 gliadin and allele-specific LMW-GS genes confirmed that these genes from the 1B chromosome are missing in DH20. The use of repeat junction primers that amplify sequences near the ends of the 6.5 Mb region of the 1B chromosome further indicated that at least a 5.8 Mb region of the 1B chromosome was deleted in the mutant line. This region of the chromosome also contains multiple resistance genes [11]. Despite the sizeable deletion and the absence of multiple prolamin genes, a previous study found that the flour from the mutant line has reasonable breadmaking quality [18].

The loss of the omega-5 gliadin genes in DH20 is of particular interest given the importance of the omega-5 gliadins in food allergies. As expected, DH20 showed reduced levels of reactivity with IgE from sera of WDEIA patients. However, it was curious that several highly immunoreactive proteins remained in this line. These proteins correspond to minor spots in 2-D gels that were found to react with a monoclonal antibody specific for omega-5 gliadins, suggesting that they are omega-5 gliadins encoded outside the deleted region of chromosome 1B.

It has been generally accepted that omega-5 gliadins are encoded by genes at the *Gli-B1* locus on the short arm of the 1B chromosome. Indeed, primers specific for the known full-length omega-5 gliadin genes amplified DNA from Chinese Spring but not from the N1BT1A or N1BT1D nullisomic tetrasomic lines. However, over the years there also have been a number of reports of omega gliadins encoded by minor loci [22, 23]. Recent genome sequence analyses in the reference wheat Chinese Spring has made it possible to survey the entire collection of gliadin and LMW-GS genes in a hexaploid wheat cultivar [11, 24]. This work uncovered four omega-5 gliadin genes on the D genome in addition to the eight on the B genome. All of the D genome genes were classified as pseudogenes because of frameshift mutations in the coding regions and low FPKM values in transcriptome data. However, one gene, omega-D4, contained only a single mutation near the 3′ end of the coding region that, if expressed, would yield a truncated protein with an altered C-terminus. It is interesting that there are a number of sequences for omega-5 gliadin genes in NCBI that encode proteins with altered C-termini. Most were from a study in which 66 omega-5 gliadins were amplified from cDNA prepared from developing grain from a variety of diploid, tetraploid and hexaploid wheats [9]. All were missing portions of the central repetitive regions and thus would encode proteins of only 4 to 38 kDa. Seventeen of the 66 sequences encoded proteins with altered C-termini similar to that of omega-D4, including 15 from the diploid species *Ae. tauschii, Ae. speltoides, Ae. searsii and T. monococcum*. It is also interesting that 23 of 29 sequences amplified from hexaploid wheat and 14 of 17 sequences amplified from tetraploid wheat encoded proteins with the N-terminal sequence SRL while all 20 of the sequences amplified from diploid wheat encoded proteins with the N-terminal sequence TRQ that is also found in omega-D4. Additionally, a gene with only 4 bp mismatch to omega-D4 was identified in *Ae. tauschii*, the D progenitor of hexaploid wheat [20].

Omega-5 gliadins are unusual in that each protein contains many copies of IgE-reactive epitopes. Omega-D4 contains a greater number of the major WDEIA epitopes than the other full-length omega-5 gliadins deposited in the NCBI database. Additionally, there is considerable overlap among these epitopes in many regions of the protein. The fact that these proteins are very minor species in both the parental lines and DH20, yet the proteins are nearly as IgE reactive with patient sera as the more abundant omega-5 gliadins suggests that both the number and the arrangement of the epitopes in the protein are likely important in determining its immunoreactivity.

In previous studies, RNA interference was used to reduce the levels of omega-5 gliadins in wheat flour, resulting in transgenic plants with reduced IgE reactivity to sera from WDEIA patients without adverse effects on flour end-use functional properties [15–17]. This homology-based approach used a 153 bp target region corresponding to the 3′ end of the coding region and effectively eliminated all omega-5 gliadins from the flour with relatively few other changes on the proteome. However, the resulting plants were transgenic and not likely to be incorporated into breeding programs because of consumer acceptance issues. The present work demonstrates that breeding approaches also can be used to reduce the levels of omega-5 gliadins and hence the immunogenic potential of the flour. However, such approaches are complicated by the genetic linkage of omega gliadin, gamma gliadin and LMW-GS genes and the finding that minor but highly immunoreactive omega-5 gliadins may be encoded on chromosomes other than 1B.

Currently, the exact sequences of omega-5 gliadins in the parental lines Keumkang and Olgeuru and the mutant line DH20 are not known. It is also not known whether other hexaploid wheat cultivars contain active omega-5 gliadin genes on either their A or D chromosomes. However, it is clear that future efforts to reduce the levels of omega-5 gliadins and other immunogenic proteins in wheat flour, either by breeding approaches that rely on induced mutations or biotechnology approaches that utilize homology-based methods such as gene silencing or genome editing, would benefit from a detailed understanding of the complete complement of gluten protein genes in individual cultivars. Clearly, it is important to know the numbers of genes and pseudogenes in these complex families as well as their precise sequences and genomic locations. To this end, the reference genome sequence from Chinese Spring now makes it possible to design gene capture methods to select genomic regions containing gluten protein genes from individual cultivars for long-read sequencing. Thus, it should be possible to obtain the complete sequences of highly repetitive omega-5 gliadin genes that have not been easily cloned in the past and survey the entire collection of these genes in different cultivars of hexaploid wheat. Such studies will also provide insight into the evolution of this interesting family of gluten protein genes.

## Conclusions

Breeding or biotechnology approaches to reduce the immunogenic potential of wheat flour require that multiple gluten proteins be eliminated without impacting the functional properties of the flour. However, the lack of complete information on the sequences of the gluten proteins and the locations of the genes that encode them complicates these efforts. In this study, a mutant wheat line missing the major omega-5 gliadins encoded on chromosome 1B was shown to have reduced IgE reactivity with sera from WDEIA patients. However, several minor but highly immunogenic proteins remained in the flour. Further analysis revealed that these proteins were omega-5 gliadins encoded by genes located on chromosome 1D that had not been described previously and that the proteins contained a greater number of WDEIA epitopes and more overlapping epitopes than those encoded on chromosome 1B. The work illustrates the importance of detailed knowledge about the complete sets of gluten protein genes and proteins in individual wheat cultivars for future efforts aimed at reducing the immunogenic potential of wheat flour.

## Methods

### Plant material

*Triticum aestivum* L. cv. Keumkang, a leading hard wheat cultivar in Korea that is used for both breadmaking and noodle production, and cv. Olgeuru, a soft wheat used for noodle production, were the parental lines used to produce a doubled haploid population from which the mutant line DH20 was selected [18]. Keumkang was shown previously to contain the *Glu-A3c*, *Glu-B3h* and *Glu-D3a* alleles while Oleguru contained the *Glu-A3d*, *Glu-B3d* and the *Glu-D3a* alleles [25]. The reference cultivar Chinese Spring, containing *Glu-A3a*, *Glu-B3a* and *Glu-D3a* alleles, and nullisomic tetrasomic lines of Chinese Spring, kindly provided by National BioResource Project-Wheat (Kyoto, Japan), served as controls for PCR analyses. Plant material was grown in the field as described previously [18].

### Protein extraction, 2-DE analysis and mass spectrometry

Total flour proteins from Keumkang, Olgeuru and DH20 were extracted with SDS buffer (2% SDS, 10% glycerol, 50 mM DTT, 40 mM Tris-Cl, pH 6.8), quantified and analyzed by 2-DE as described previously [19]. Individual protein spots were excised from Coomassie-stained gels and transferred to 96-well plates for digestion with thermolysin, chymotrypsin or trypsin using a DigestPro (Intavis, Koeln, Germany). Plates containing the peptides were placed in the autosampler of a nanoflow HPLC interfaced to an Orbitrap Elite mass spectrometer (Thermo Scientific, San Jose, CA). Details of data collection are described in [26]. MS/MS spectra were searched against a database containing *Triticeae* protein sequences downloaded from NCBI on November 1, 2016, cultivar-specific sequences from *Triticum aestivum* cv. Butte 86 reported in [19] and a database of common contaminants downloaded on January 30, 2015. Two search engines, Mascot and X!Tandem, were used for the analyses and the results were compiled in Scaffold Version 4.7.5 (Proteome Software, Inc., Portland, OR). Data extracted from Scaffold are summarized in Additional file 1.

An updated database was used for the identification of minor IgE reactive proteins in DH20. This database included *Triticeae* protein sequences downloaded from NCBI on February 7, 2018, cultivar-specific gluten protein sequences from cv. Butte 86 [19], sequences of gliadins and LMW-GS from cv. Chinese Spring [11, 24] and a database of common contaminants downloaded on January 30, 2015.

### PCR analysis

Genomic DNA was isolated from young leaf tissue of Keumkang and Olgeuru parental lines, DH20 mutant line, Chinese Spring, and the nullisomic tetrasomic lines of Chinese Spring using the DNeasy Plant mini kit (Qiagen, Hilden, Germany) according to protocols provided by the manufacturer. PCR was performed using Extaq polymerase (Takara Bio Inc., Kyoto, Japan). PCR primers and amplification conditions are shown in Table 2. Primers specific for LMW-GS *GluB3–4* gene found in Keumkang, Olgeuru and Chinese Spring were described previously [27]. Forward and reverse primers for omega-5 gliadin were based on the sequence of AB181300 and would be expected to amplify a 1212 bp fragment. The design of repeat junction primers is described in [28]. These primers were based on the assembled sequence of a 6.5 Mb region of chromosome 1B from cv. Chinese Spring reported in [11], NCBI Accession Number MG560141. Primer pair 19S-1.3-2 amplifies a 418 bp region that is 1.74 Mb upstream of the first ancestral gene shown in Fig. 2 in [11]. The forward primer corresponds to the sequence between position 700,904 and 700,923 and the reverse primer corresponds to the sequence between 701,298 and 710,321 in NCBI Accession Number MG560141. Primer pair 143E-1-600 amplifies a 482 bp region that is just before the last two ancestral genes shown in Fig 2 in [11]. The forward primer corresponds to the sequence between 6,512,265 and 6,512,288 and the reverse primer corresponds to the sequence between 6,512,722 and 6,512,746 in NCBI Accession Number MG560141.

PCR primers were also designed to amplify the omega-D4 gene from cv. Chinese Spring reported in [11]. The forward primer corresponds to the sequence from 58 to 78 bp downstream from the initiation codon and the reverse primer corresponds to the sequence from 1074 to 1094 downstream from the initiation codon. The forward and reverse primers have three and seven base mismatches with AB181300, the gene sequence encoding omega-5 gliadin BAE20328.

### Immunoblot analysis

For immunoblot analysis of the parental and mutant lines, 7.5 μg total SDS-extracted protein were separated by 2-DE and transferred onto nitrocellulose membranes as described in [29]. Immunoblot analysis was performed as described in [30] using sera from WDEIA patients that was obtained with the informed consent of patients and approval from the Ethics Committee of Ile de France III and the French Agency for the Safety of Heath Products (AFSSAPS) (authorization number 2008-A01565–50) and characterized previously [16]. IgE-binding proteins were visualized using peroxidase-labeled rabbit anti-human IgE (9160–05 SouthernBiotech) diluted 1:500,000 and chemiluminescent substrate (WesternBright Quantum, Advansta K-120420D20) according to the manufacturer’s instructions. Alternately, membranes were reacted with a monoclonal antibody against the peptide SRLLSPRGKELG (mono ONT 18A5) that was diluted 1/80 and visualized using peroxidase-labeled goat anti mouse IgG (H, L)-HRP conjugate (170–6516 Biorad) diluted 1:50,000.

## Additional file

Additional file 1: Summary of MS/MS data from protein spots shown in Fig. 1. Data was extracted from Scaffold. (XLSX 3032 kb)

## Abbreviations

2-DE: Two-dimensional gel electrophoresis
MS/MS: Tandem mass spectrometry
WDEIA: Wheat dependent exercise-induced anaphylaxis
